# Advancing Women’s Performance in Fitness and Sports: An Exploratory Field Study on Hormonal Monitoring and Menstrual Cycle-Tailored Training Strategies

**DOI:** 10.3390/sports14010007

**Published:** 2026-01-01

**Authors:** Viktoriia Nagorna, Kateryna Sencha-Hlevatska, Daniel Fehr, Mathias Bonmarin, Georgiy Korobeynikov, Artur Mytko, Silvio R. Lorenzetti

**Affiliations:** 1 Swiss Federal Institute of Sport Magglingen, 2532 Magglingen, Switzerland; 2 Sport Games Department, National University of Ukraine on Physical Education and Sport, 03150 Kyiv, Ukraine; 3 Chuiko Institute of Surface Chemistry NAS of Ukraine, 03164 Kyiv, Ukraine; ksencha.h@gmail.com; 4 School of Engineering, ZHAW Zurich University of Applied Sciences, 8401 Winterthur, Switzerlandsl@ethz.ch (S.R.L.); 5 Department of Theory and Methodology of International Types of Wrestling, Uzbek State University of Physical Education and Sport, Chirchik 111700, Uzbekistan; k.george.65.w@gmail.com; 6 Department of Health Sciences and Technology, ETH Zurich, 8092 Zurich, Switzerland

**Keywords:** women’s health, hormonal influences, fitness and sports, hormone monitoring, female athletes, public health

## Abstract

Background. Extensive research confirms that hormonal fluctuations during the menstrual cycle significantly influence female athletic performance, with profound implications for public health, including promoting equitable access to sports and enhancing women’s overall physical and mental well-being. Numerous scientifically validated methods are available to monitor hormonal status and menstrual cycle phases. However, our prior investigations revealed that these insights are rarely applied in practice due to the complexity and invasiveness of existing methods. This study examines the effects of hormonal fluctuations on elite female basketball players. It assesses practical, non-invasive, cost-effective, and field-applicable methods for hormonal monitoring, with a focus on cervical mucus analysis for estrogen crystallization. The goal is to optimize training, promote equity in women’s sports, and support public health strategies for female empowerment through sustained physical activity, addressing the limitations of male-centric training models. Materials and Methods. This exploratory field study employed a multifaceted approach, beginning with a comprehensive meta-analysis via literature searches on PubMed, SCOPUS, and Google Scholar to evaluate hormonal impacts on physical performance, supplemented by an expert survey of 20 sports scientists and coaches using Kendall’s concordance coefficient for reliability and an experimental phase involving 25 elite female Ukrainian basketball players assessed over three months through daily performance tests (e.g., sprints, jumps, agility drills, and shooting) integrated into six weekly training sessions, with cycle phases tracked via questionnaires, basal body temperature, and the fern leaf method for estrogen levels. Results. Performance peaked during the postmenstrual and post-ovulatory phases (e.g., a 7.5% increase in sprint time and a 5.1% improvement in running jump). It declined in the premenstrual phase (e.g., a 2.3% decrease in acceleration). The estrogen crystallization test using cervical mucus provided preliminary insights into hormonal status but was less precise than laboratory-based methods, such as LC-MS/MS, which remain impractical for routine use due to cost and complexity. The fern test and basal body temperature showed limited precision due to external factors. Conclusions. There is a critical need to develop simple, non-invasive, field-applicable devices for accurate, real-time hormonal monitoring. This will bridge the gap between research and practice, enhancing training personalization, equity in women’s fitness and sports, and public health outcomes by increasing female participation in physical activities, reducing gender-based health disparities, and fostering inclusive wellness programs.

## 1. Introduction

Physical activity, defined as any bodily movement produced by skeletal muscles that results in energy expenditure, is critical for physically active women, particularly female athletes, who must understand their hormonal status to optimize physical and cognitive performance during training and competition [1]. Hormonal fluctuations across the menstrual cycle significantly influence strength, endurance, coordination, and recovery, necessitating tailored training approaches to enhance performance and reduce injury risk [1,2,3,4,5,6,7]. Despite the growing participation of women in elite sports, the unique physiological demands associated with these hormonal changes remain underexplored, as sports science has historically been dominated by male-centric training models [1,3]. This male-oriented approach often overlooks phase-specific physiological mechanisms in women, which, when addressed, can improve performance outcomes, such as leveraging peak power during the postovulatory phase or adjusting intensity during menstruation [3,6,7].

While the importance of monitoring hormonal levels is widely acknowledged among coaches and athletes, practical implementation remains limited due to perceived inconveniences and complexities, as identified through prior expert interviews [3]. Conventional hormonal control methods, primarily designed for male athletes, fail to account for the dynamic hormonal profiles of female athletes, leading to suboptimal training regimens [3,5]. Recent research has highlighted that aligning exercise with menstrual cycle phases can enhance performance; however, the lack of accessible, practical, and non-invasive monitoring methods hinders the widespread adoption of this approach [8,9,10].

Hormonal fluctuations in women, primarily driven by dynamic changes in progesterone and estrogen, define critical phases of the menstrual cycle: the follicular phase, ovulation, and luteal phase [11]. Estradiol, a primary estrogen, peaks during the follicular phase, triggering the luteinizing hormone (LH) surge that initiates ovulation. Progesterone then rises post-ovulation to support the luteal phase [11,12]. These hormones significantly influence physical activity. Studies have shown that elevated estradiol levels during the follicular phase enhance muscle strength, power output, and endurance. In contrast, progesterone in the luteal phase may increase body temperature and cardiovascular strain, potentially reducing exercise capacity [11,12,13]. These effects can be modulated by factors such as Relative Energy Deficiency in Sport (RED-S) and dietary patterns (e.g., carbohydrate availability influencing estrogen levels), with implications for injury prevention, including increased ACL risk during certain phases. Hormonal fluctuations also affect male physical activity, though less markedly, with testosterone levels, which vary by time of day and lifestyle factors, influencing muscle mass, strength, and recovery [14,15,16,17]. Understanding hormonal status is thus crucial for both genders to optimize training regimens and performance outcomes.

Timely detection of hormonal fluctuations is particularly vital for physically active women, as it enables personalized training plans, supports fertility tracking, and aids in diagnosing reproductive disorders such as polycystic ovary syndrome (PCOS) [18]. The development of accessible and reliable monitoring methods is an urgent task, as it addresses health, performance, and equity issues in sports. Hormonal variations across the menstrual cycle influence strength, endurance, and recovery, yet male-centric training models often overlook these dynamics, leading to suboptimal outcomes for women [1,19]. Non-invasive monitoring can facilitate tailored exercise programs, reduce the risks of overtraining, and enhance recovery in high-performance contexts, such as sports, military training, and rehabilitation [20,21,22,23,24].

Currently, a variety of methods have been developed for the accurate detection of female sex hormones. The most frequently used assay method in the European Pharmacopoeia remains UV spectroscopy, whereas in the United States, high-performance liquid chromatography (HPLC) is used [22]. Other methods, such as electroanalytical, fluorescence, and Raman spectroscopy [20,22,24,25,26,27], have also been explored. Immunoassay (IA) methods play a significant role because they are non-invasive, require minimal laboratory conditions, do not require specialized personnel skills, and are cost-effective. Nevertheless, IAs have drawbacks, including a lack of specificity, sensitivity, cross-reactivity with other hormones and metabolites, and matrix effects. Moreover, IAs show poor performance in analyzing samples from men, children, and postmenopausal women [28]. The best results in the analysis of E2 and P4 have been achieved using HPLC separation combined with mass spectrometry (MS) [24,25,26,28]. Using MS analysis, more than 20 different hormones can be detected simultaneously. However, the MS method requires long-term sample preparation, including extraction, separation, and concentration via high-performance liquid chromatography (HPLC) with highly toxic solvents or via solid-phase extraction (SPE) on adsorbents [14]. Moreover, while MS is desirable in clinical practice, it remains the most expensive method, requiring the purchase of an MS instrument and specialized technician training [25]. The colorimetry method (CM), based on color chemical reactions, could also be used. Some useful color reactions, such as the condensation of steroids with isoniazid, indirect measurements after reaction with Tetrazolium Blue and phenylhydrazine, and Friedel-Crafts acylation, have been undeservedly forgotten [25]. Progesterone can be detected through its reaction with dinitrophenylhydrazine or m-dinitrobenzene [13]. Modern visual methods are more complex but also more sensitive and selective. Based on gold nanoparticles (AuNPs) and DNA aptamers, color changes can be observed in the presence of E2 or P4 [24,25,27,28]. Apart from AuNPs-based colorimetric assays, a colorimetric sensor based on the catalytic reaction of 3,3′,5,5′-tetramethylbenzidine (TMB) and a peroxidase substrate was also developed for monitoring E2/P4 indirectly [29].

Existing methods for detecting a woman’s hormonal status can be broadly categorized into four main groups (Table 1).

Invasive methods, such as blood draws, remain the gold standard for hormone measurement due to their high accuracy and ability to detect total hormone levels [30,31]. Liquid chromatography–tandem mass spectrometry (LC-MS/MS) applied to blood samples offers high specificity and multi-hormone profiling, making it an ideal tool for clinical diagnostics [25,26]. However, these methods are costly, require specialized equipment and trained personnel, and cause discomfort, rendering them impractical for frequent or real-time monitoring in athletic settings [14,28]. These limitations drive the demand for non-invasive alternatives that are more practical for routine use. Non-invasive methods, including those that utilize saliva, urine, sweat, and interstitial fluid (ISF) sensing, offer promising alternatives. Saliva-based monitoring correlates well with free, bioavailable hormone levels in blood. It is enhanced by electrochemical sensors that utilize nanomaterials, such as graphene and gold nanoparticles, achieving detection limits in the picomolar range [13,20,26]. These sensors offer portability and rapid results, making them suitable for exercise contexts. Wearable devices with wireless data transmission enable continuous monitoring [22]. ISF sensing, which measures hormones in the fluid surrounding cells using microneedle-based patches, offers high sensitivity and real-time capabilities [31]. Membrane-based preconcentration techniques further improve detection by concentrating analytes for analysis [32]. However, challenges include lower hormone concentrations in saliva and ISF compared to blood, variability due to saliva flow rates or contamination, and concerns about sensor durability during intense physical activity [30,33]. Optical methods, such as fluorescence and colorimetry on paper-based platforms, are cost-effective and field-adaptable; however, they lack precision for quantitative analysis and are susceptible to environmental interference [24,25]. Immunoassays, valued for simplicity, suffer from cross-reactivity and reduced sensitivity in specific populations, such as postmenopausal women [28].

This study addresses these challenges by evaluating the practicality and effectiveness of various hormonal monitoring methods for female athletes. By examining both invasive and non-invasive techniques, including salivary hormone testing, wearable sensors, and point-of-care devices, we aim to identify approaches that are user-friendly, reliable, and feasible for regular use in athletic settings [13,20,21,22,24,25,27,28,29]. Our research seeks to bridge the gap between the recognized importance of hormonal monitoring and its practical application, offering insights to optimize training for female athletes and address the limitations of male-centric sports science paradigms.

Currently, no single hormonal monitoring method combines convenience, accuracy, and scalability for routine use. Developing such a method is a critical and promising task, with significant implications for improving the health, performance, and reproductive well-being of physically active women, as well as supporting the health of future generations. In this context, this study aims to practically demonstrate the application of existing hormonal monitoring methods and their impact on specialized physical performance in female athletes, using elite basketball players as a case study, and to analyze prospects for improving these methods through modern knowledge and innovative technologies.

Although the effects of menstrual cycle phase on female athletic performance are well documented, the reasons why this knowledge is rarely applied in daily training remain unclear. The present exploratory field study is the first to systematically evaluate the real-world usability of the most recommended low-cost non-invasive monitoring methods in a highly controlled professional setting (elite female basketball players, *n* = 25, 3 months of testing). The deliberately homogeneous, professional sample was chosen to test these methods under the most favorable conditions possible. The fact that they still proved impractical in this context strongly indicates that the identified barriers are universal for all physically active women, from recreational fitness enthusiasts to elite individual-sport athletes.

While male-centric models dominate, this study focuses exclusively on novel practical barriers to implementing women-specific strategies, rather than direct sex comparisons.

## 2. Materials and Methods

### 2.1. Meta-Analysis

To conduct a comprehensive meta-analysis, a literature review examined the impact of hormonal fluctuations on physical performance in sports and exercise science. The search was conducted across multiple databases, including PubMed (MEDLINE), SCOPUS, and Google Scholar. Articles were selected based on title relevance and full texts related to physical performance assessments. The meta-analysis was conducted in accordance with the PRISMA (Preferred Reporting Items for Systematic Reviews and Meta-Analyses) guidelines.

### 2.2. Expert Survey

Additionally, an expert survey was conducted involving 20 sports scientists and national team coaches experienced in working with elite female athletes. Experts identified contemporary challenges in training high-achieving women in sports. Kendall’s concordance coefficient (W) was used to quantify agreement among experts, and the normative coefficient of significance (α) was determined to ensure the reliability and validity of the collected data.

### 2.3. Experimental Part (Participants and Procedure)

This study involved 25 professional female basketball players competing in the Ukrainian Super League and Higher League. The participants were:

- Super League team *FRANKIVSK-PRYKARPATTIA* (Ivano-Frankivsk region): 18 players with a mean age of 21.3 ± 5.3 years.
- Higher League team *FRANKIVSK-PNU-DYUSSH-2* (Ivano-Frankivsk): 7 players with a mean age of 21.29 ± 7.8 years.

All participants, and a parent/guardian for those under 18, provided written informed consent for their data to be used for research purposes.

The athletes had an average height of 177.14 ± 6.3 cm and an average weight of 69.14 ± 6.5 kg. Their role distribution was as follows: forwards (57%), guards (28%), centers (14%). All participants were in good health and provided informed consent before participation, under the study protocol approved by the local Biomedical Ethics Committee (2 June 2025, protocol No. 5). All athletes were assessed for baseline physical condition using standard tests, including VO_2_max (mean 48.5 ± 3.2 mL/kg/min) and body composition (body fat 18.2 ± 2.1% via DEXA), confirming elite-level fitness.

The experiment spanned three months, with athletes participating in six training sessions per week (Monday to Saturday), and Sundays designated as rest days. The study was conducted during the off-season (June–August 2025), with no official matches scheduled, allowing focus on controlled training sessions without game-related fatigue or scheduling conflicts. The study assessed the impact of hormonal variations during the menstrual cycle on performance using the following methods:

- Questionnaires: To collect subjective data on athletes’ menstrual cycles and training perceptions.
- Basal Body Temperature Measurement: To track ovulatory and menstrual cycle phases [34,35].
- Estrogen Level Determination: Conducted using the “fern leaf” method to identify cycle phases [36].
- Performance Testing Equipment:

○ Video analysis tools for sprint and movement assessment.
○ Digital timers and stopwatches for precise time measurement.
○ Jump height measurement devices for vertical jump assessments.
○ Weighted medicine balls (2 kg) for strength testing.
○ Court markings and obstacle placements for agility and endurance evaluations.

Performance tests were conducted daily during each of the six weekly training sessions to evaluate performance indicators critical to athletic development, including speed, agility, power, endurance, and technical precision. Tests were integrated at the beginning of each training session to minimize the impact of accumulated fatigue, with a standardized 10 min warm-up before testing. Tests were integrated into regular training to reduce disruption while ensuring comprehensive data collection. The testing schedule is summarized below (Table 2):

This schedule ensured balanced assessment: for example, agility was tested three times per week (Monday, Wednesday, Friday), acceleration twice (Monday, Friday), and strength twice (Monday, Thursday), allowing for recovery while capturing daily fluctuations.

A key test included:

- Acceleration Test: This measured sprinting efficiency over 20 m from a stationary position, with an intermediate time recorded at the 6 m mark. Video analysis assessed the first-step technique, which involves the biomechanics of the initial sprint step, including foot placement, body lean, and force application, to optimize sprint initiation and efficiency.
- Sprint with Deceleration and Change of Direction: This required athletes to sprint 20 m at ≥95% of maximum speed, execute a controlled braking maneuver, stop, and reverse back to the starting line.
- Standing Vertical Jump with Target Reach: Athletes jumped to touch the highest point on the backboard, with active arm motion and stationary arms.
- Running Vertical Jump with One-Leg Takeoff: Athletes executed an approach jump from the three-second zone with a one-leg takeoff, analyzed via height-to-weight ratio coefficient.
- Jumping Speed Test: Athletes performed consecutive jumps over obstacles in a cross pattern, with jumps counted within a 20 s time frame.
- Speed Dribbling Technique Test: Athletes maneuvered a basketball through a slalom course with three obstacles, finishing with a lay-up shot.
- Medicine Ball Throw for Distance and Accuracy: Athletes threw a 2 kg ball using a single-handed shoulder pass from stationary and running positions within a 2 m wide corridor.
- Defensive Movement Speed and Agility Test: Athletes sprinted from the baseline to five points at the three-point line corners using forward, backward, and lateral movements.
- Specific Endurance Test: Athletes performed a shuttle run across the court (backboard to backboard) with backboard touches, completing three sets of five repetitions with 30 s rest intervals.
- Mid-Range and Long-Range Shooting Consistency Test: Athletes attempted shots from ten locations (five at 4.5 m, five at 6.25 m).
- Free Throw Consistency Test: Athletes attempted 20 free throws, alternating backboards with dribbling sequences.

### 2.4. Statistical Analysis

This study employed a comprehensive statistical approach to analyze the influence of hormonal fluctuations across the ovulatory-menstrual cycle on the physical and technical performance of elite female basketball players (*n* = 25). Data normality was assessed using the Shapiro–Wilk test to confirm the appropriateness of parametric statistical methods for each performance metric (e.g., sprint times, jump heights, shooting consistency) and hormonal measurements (e.g., estrogen levels via the fern test). Levene’s test was applied to evaluate the assumption of equal variances across menstrual cycle phases (menstrual, postmenstrual, ovulatory, post-ovulatory, premenstrual). These preliminary checks ensured the validity of subsequent analyses by confirming that the data met the assumptions for parametric testing.

To compare performance metrics across menstrual cycle phases, a one-way repeated-measures analysis of variance (ANOVA) was used to assess differences in means for each test (e.g., 20 m sprint, vertical jump, shooting consistency) across the five phases of the cycle. Post hoc pairwise comparisons were conducted using Bonferroni corrections to identify specific phase differences while controlling for Type I error. For hormonal data, paired *t*-tests were employed to compare estrogen crystallization levels (e.g., “+”, “++”, “+++”) between consecutive menstrual cycle phases, ensuring precise tracking of hormonal fluctuations.

When normality assumptions were not met, nonparametric alternatives, such as the Friedman test for repeated measures or the Wilcoxon signed-rank test for paired comparisons, were used to ensure robust analysis. However, the Shapiro–Wilk test indicated that most performance and hormonal datasets were normally distributed, supporting the use of parametric methods.

Correlation analyses were conducted to explore relationships between hormonal fluctuations and performance outcomes. Spearman’s rank correlation coefficient (ρ) was used to assess the associations between estrogen crystallization levels and performance metrics (e.g., sprint speed, jump height), given the ordinal nature of the fern test results. Additionally, Kendall’s concordance coefficient (W) was used to quantify the agreement among expert survey responses (*n* = 20 sports scientists and coaches) regarding training challenges for female athletes, with a significance level (α) set at 0.05 to ensure reliability.

The study maintained a 95% reliability level (*p* < 0.05) to ensure statistically robust findings. Power analysis was conducted a priori to determine the required sample size for detecting phase-specific performance differences. Assuming a large effect size (Cohen’s d = 0.8), an alpha error probability of 0.05, and a power of 0.80 (1-β error probability), a minimum sample size of 20 participants was deemed sufficient, confirming that the study’s sample (*n* = 25) was adequately powered. All statistical analyses were performed using SPSS (Version 28.0), and data visualizations were generated to illustrate phase-specific trends and correlations.

This statistical framework ensured methodological rigor, enabling accurate interpretation of the relationships among menstrual cycle phases, hormonal fluctuations, and athletic performance, while providing a robust foundation for developing evidence-based, gender-specific training strategies.

## 3. Results

### 3.1. Meta-Analysis of Methods for Determining the Hormonal Status Level in Women Engaged in Physical Activity

**Figure 1:**
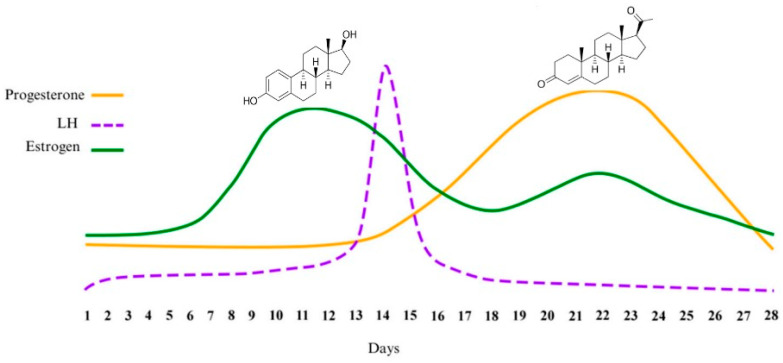
Hormonal Fluctuations of Progesterone, LH, and Estrogen Across the Menstrual Cycle.

Accurate determination of the hormonal profile remains a challenging task, despite all efforts devoted to this problem. Methods such as counting cycle days, luteinizing hormone testing, and body temperature are easy but may be inadequate when used as the sole method of verification [35]. The “fern test” can only indicate the periovulatory period, but not a precise phase [35,36,37]. From a chemical perspective, during the menstrual cycle (26–31 days), two primary sex hormones are prevalent: 17β-estradiol (E2) and progesterone (P4) (Figure 1, adapted from [35]).

During the menstrual cycle, the concentrations of estrogen (E2) and progesterone (P4) change dramatically. Due to significant differences in hormonal composition across the various phases, simultaneous analysis of E2 and P4 enables precise determination of the menstrual cycle (MC) phase. However, the relatively low values of E2 and P4 (pg/mL), as well as their cross-reactivity in matrices such as blood, urine, saliva, or tissues, make their detection challenging [35,37]. Blood plasma, urine, or saliva are typically used as biological materials for detecting sex hormone levels [30]. The most appropriate material for the accurate quantitative determination of E2 and P4 is blood plasma. However, this invasive method requires specialized sampling conditions and is time-consuming for sample preparation before analysis. Urine collection is a non-invasive and straightforward method; however, urine contains numerous metabolites that can interfere with the determination of target substances. The concentration of E2/P4 in saliva is the lowest; however, a significant linear correlation was observed between E2/P4 levels in saliva and blood serum [38]. Additionally, frequent saliva collection is convenient, stress-free, and inexpensive.

The meta-analysis presented in Table 3 provides a comprehensive evaluation of non-invasive methods for hormone detection, offering critical insights for tracking hormonal profiles, particularly in sports contexts. Urine-based devices, such as Mira and Inito, demonstrate superior performance for at-home phase detection, with high accuracy (95–99%), rapid result delivery (10–20 min), and affordability ($150–260) (Hackney, 2021; Higashi, 2012) [17,25]. These devices measure estrone-3-glucuronide (E3G), luteinizing hormone (LH), pregnanediol glucuronide (PdG), and follicle-stimulating hormone (FSH), enabling precise identification of follicular and luteal phases. Integration with Internet of Things (IoT) and artificial intelligence (AI) enhances usability by providing personalized insights, making these devices highly suitable for real-time monitoring in athletic settings. Wearable aptamer nanobiosensors exhibit exceptional sensitivity for continuous estradiol monitoring (limit of detection [LOD] 0.14 pM). However, they are limited by their inability to detect progesterone, restricting their utility for comprehensive menstrual cycle tracking (Wang et al., 2023) [39]. Liquid chromatography-tandem mass spectrometry (LC-MS/MS) remains the gold standard for hormone profiling, achieving accuracy levels of 86.5–98%. However, its high cost and prolonged processing time render it impractical for real-time applications (Miyachi et al., 2017) [40].

Despite the availability of various commercial products, no convenient, validated, point-of-care method currently exists for the simultaneous detection of estrogen and progesterone suitable for sports applications. Biosensors that measure electrical signals from sensitive reactions to low concentrations of hormones show significant promise. However, these technologies remain in the research and development (R&D) phase. Furthermore, such devices can be integrated with IoT and AI, enabling data interpretation and the development of personalized training recommendations.

The survey of 20 experienced sports scientists and coaches showed strong agreement regarding the main barriers to implementing menstrual cycle-based training (Kendall’s W = 0.81, *p* < 0.001). The highest-ranked barriers were “lack of simple and reliable field monitoring methods” (85% of experts ranked it 1–2) and “difficulty of individualizing training in team sports when athletes are in different phases” (75% ranked 1–2).

### 3.2. Experimental Section: Practical Implementation of Hormonal Status Monitoring in Elite Female Athletes

In sports science, direct methods for assessing hormonal fluctuations, such as transvaginal follicular scanning, are rarely used due to their invasiveness, high cost, and time requirements [1]. Instead, indirect methods, such as questionnaires, basal body temperature (BBT) measurements, and calendar-based cycle tracking, are commonly employed [35]. In this study, we utilized these methods because they are accessible, widely used in sports research, and supported by prior studies [24,25,27,28,35]. However, menstrual cycle phases determined by these methods do not always accurately reflect the hormonal profile, potentially compromising the reliability of the results [11,20]. For instance, calendar-based tracking cannot distinguish between ovulatory and anovulatory cycles, and BBT is sensitive to external factors such as stress or sleep disturbances [35,41].

To assess estrogen levels, we applied the “fern test,” a microscopic technique that evaluates estrogen saturation by analyzing the crystallization patterns of dried cervical mucus [35,36]. This method detects changes in mucus structure driven by hormonal fluctuations throughout the menstrual cycle [36]. During the postmenstrual phase, estrogen crystallization was initially weak (“+”), progressively increasing to “+++” before the ovulatory phase in most participants. However, two athletes reached a maximum of only “++” crystallization, suggesting moderate estrogen saturation [37]. A “+++” result indicates peak estrogen levels, typically observed around ovulation, while a “++” result reflects partial crystallization, indicating moderate estrogen levels, which may be normal in the follicular or luteal phases. A “+” or absent crystallization suggests low estrogen levels, common in the post-ovulatory phase or conditions like hypoestrogenism [35].

The fern test provides a preliminary indication of estrogen levels but is not a precise diagnostic tool [37]. For accurate assessment, laboratory blood tests for estradiol, typically conducted on days 2–5 of the cycle, remain the gold standard [33,37]. Additionally, evaluating menstrual irregularities (e.g., amenorrhea, oligomenorrhea) or symptoms such as mucosal dryness, reduced libido, fatigue, or deteriorating skin and hair condition can help identify potential estrogen imbalances [30]. In conclusion, while the fern test offers valuable insights into estrogen saturation, a “++” result alone does not confirm deficiency. A comprehensive evaluation, including blood tests and clinical assessments, is essential for accurate diagnosis and effective training planning in elite female athletes.

The experimental group’s menstrual cycle ranged from 25 to 31 days, with an average cycle length of 28.12 days. The cycle was divided into phases based on fluctuations in sex hormone concentrations: menstrual, postmenstrual, ovulatory, post-ovulatory, and premenstrual. Estrogens, progesterone, and androgens played key adaptive and trophic roles, exerting anabolic effects. The influence of androgens on anabolic processes was more pronounced than that of estrogens and progesterone, contributing to the regulation of metabolic and physiological responses during training and competition.

To achieve the objectives of our study, a series of specialized tests was conducted to evaluate the performance of 25 elite female basketball players competing in the Super League and Higher League.

**Figure 2:**
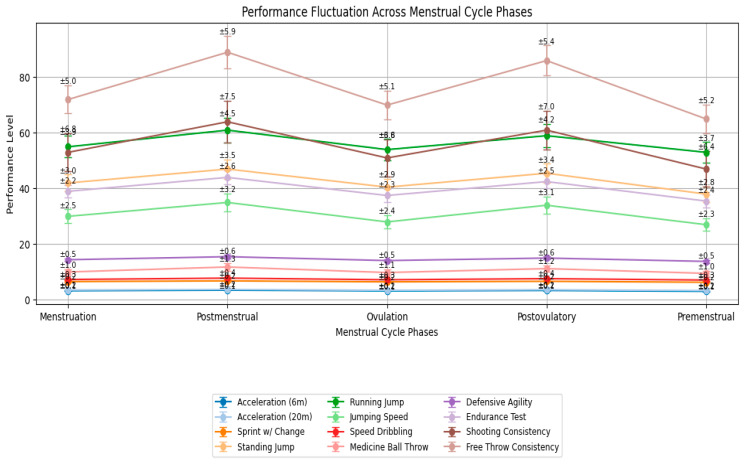
Performance Fluctuations Across Menstrual Cycle Phases in Elite Female Basketball Players.

A series of specialized tests was conducted to evaluate the performance effectiveness of 25 elite female basketball players competing in the Super League and Higher League. The average results of these tests provide critical insights into their physical and technical capabilities (Figure 2):

- Acceleration Test (20 m sprint): 3.12 ± 0.15 s (6 m), 3.28 ± 0.20 s (full 20 m).
- Sprint with Deceleration and Change of Direction: 6.74 ± 0.25 s.
- Standing Vertical Jump with Target Reach: 42.5 ± 3.2 cm.
- Running Vertical Jump with One-Leg Takeoff: 58.7 ± 4.1 cm (height-to-weight ratio coefficient).
- Jumping Speed Test: 32.4 ± 2.8 jumps in 20 s.
- Speed Dribbling Technique Test: 7.68 ± 0.35 s.
- Medicine Ball Throw for Distance and Accuracy: 10.8 ± 1.1 m.
- Defensive Movement Speed and Agility Test: 14.92 ± 0.55 s.
- Specific Endurance Test (shuttle run): 41.7 ± 2.4 s (three sets).
- Mid-Range and Long-Range Shooting Consistency: 63.4 ± 7.2%.
- Free Throw Consistency: 81.6 ± 5.4%.

This comprehensive testing protocol, along with hormonal and psychophysiological control, effectively assesses key physical and technical performance indicators to optimize the training effectiveness of elite female basketball players. The results provide valuable benchmarks that can inform coaching strategies and individual athlete development, ensuring a structured approach to enhancing overall team performance.

A total of 1827 individual performance tests were collected from 25 elite female basketball players over three complete menstrual cycles. Data were grouped into five phases: Menstruation, Postmenstrual (early follicular), Ovulation (±2 days), Postovulatory (mid-late follicular/early luteal), and Premenstrual (late luteal).

Repeated-measures ANOVA revealed statistically significant effects of menstrual cycle phase on most performance variables (Table 3, Figure 2).

#### Key Statistical Results

- Acceleration 6 m: F(4,96) = 7.89; *p* < 0.001; η^2^p = 0.25. Post Hoc (Bonferroni): Postovulatory vs. Premenstrual: −2.3% (*p* = 0.012).
- Sprint with Change of Direction: F(4,96) = 14.62; *p* < 0.001; η^2^p = 0.38. Post Hoc: Postovulatory vs. Premenstrual: −7.5% (*p* < 0.001); Postmenstrual vs. Premenstrual: −6.8% (*p* = 0.002).
- Running Vertical Jump: F(4,96) = 10.34; *p* < 0.001; η^2^p = 0.30. Post Hoc: Postovulatory vs. Premenstrual: +5.1% (*p* = 0.003).
- Speed Dribbling: F(4,96) = 9.11; *p* < 0.001; η^2^p = 0.28. Post Hoc: Postovulatory phase fastest (−4.2% vs. Premenstrual, *p* = 0.008).
- Shooting Consistency (mid-range + long-range): F(4,96) = 5.67; *p* = 0.003; η^2^p = 0.19. Post Hoc: Postovulatory +4.7% vs. Menstruation (*p* = 0.021).
- Defensive Agility and Medicine Ball Throw showed trends toward improvement in the postovulatory phase but did not reach statistical significance (*p* = 0.062 and *p* = 0.079, respectively).

The most significant and most consistent performance peaks occurred in the postovulatory and postmenstrual phases, whereas the lowest values were systematically observed in the premenstrual phase.

Despite daily application of three low-cost non-invasive methods (basal body temperature, cervical mucus ferning, and menstrual cycle questionnaires):

Only 63% of expected ovulations were correctly identified by the fern test (compared with self-reported mid-cycle symptoms used as reference).

Basal body temperature provided reliable confirmation of ovulation in only 58% of cycles, due to external factors (training time, sleep disruption, ambient temperature).

Combined use of all three methods yielded correct phase identification on ≥80% of training days in only 11 of 25 athletes.

These findings demonstrate that currently recommended low-cost methods are insufficiently reliable for daily training individualization in a professional team-sport setting.

## 4. Discussion

Our analysis confirmed findings from prior research [1,11,12,15,17,35,36] and broadened our understanding of how hormonal fluctuations across the ovulatory-menstrual cycle impact female athletes during sports training. Key findings include significant performance variations, with peak levels observed during the postmenstrual and post-ovulatory phases (e.g., +7.5 for sprint change, +5.1 for running jump), and declines in the premenstrual phase (e.g., +2.3 for acceleration 6 m). The practical implementation of hormonal status monitoring revealed challenges in applying popular methods, such as the “fern test,” BBT, and questionnaires, in daily training and competition settings, due to their susceptibility to external influences and limited real-time accuracy [37]. These results underscore that menstrual cycle phases significantly influence female athletic performance. The experimental data align well with studies by McNulty et al. (2020) [11] and Meignié et al. (2021) [15], allowing for the development of tailored training programs that optimize performance based on individual hormonal profiles. Further integration of non-invasive, reliable monitoring techniques is needed to enhance daily athletic management.

Integrating psychophysiological, anthropometric, and hormonal analyses provides a comprehensive understanding of the unique physiological and hormonal profiles of female athletes. These insights can profoundly inform the design of tailored training programs to maximize female athletes’ performance and well-being.

By combining knowledge of hormonal fluctuations across the ovulatory-menstrual cycle with gender-specific physiological characteristics, training regimens can be strategically adjusted to leverage the varying attributes of the menstrual cycle phases. For instance, during the menstruation phase, when reduced muscle strength and speed are prominent, training activities could be modified to focus on short-term work capacity while considering the potential impact of depression and indifference.

Recognizing gender-specific differences in neural processes, mobility, strength, balance, and postural stability underscores the need for personalized training approaches. Given the distinct physiological advantages of female athletes, emphasizing speed-strength exercises and focusing on coordination and balance training could be particularly beneficial.

Furthermore, understanding the peak working capacity during the post-ovulatory phase and the reduced coordination abilities and work capacity during the premenstrual phase can aid in optimizing the timing and intensity of training sessions throughout the menstrual cycle, thereby enhancing overall performance and minimizing the risk of injury. Integrating effective methods for determining the phases of the ovulatory-menstrual cycle in women engaged in fitness and sports into the planning and execution of training programs can yield more efficient, customized approaches to enhance athletic performance and women’s health.

This study makes a significant contribution to the ongoing discourse on gender-specific characteristics in elite athlete training, with a particular focus on speed and strength performance. Persistent disparities in sports science provisions and infrastructure persist between male and female athletes, limiting access to critical support and hindering optimal performance for women [35]. This gap underscores the urgent need for customized training protocols tailored to the distinct physiological and biomechanical needs of female athletes. Our findings align with established research, confirming significant gender differences across multiple dimensions of sports preparedness, including anthropometric variations, physical performance indicators, physiological characteristics, and movement demands [12,16,35].

Specifically, our work corroborates studies that highlight differences in skeletal muscle composition, maximum velocity, and strength, which consistently show lower values in female athletes than in males [19]. Additionally, our research underscores the significant impact of the ovulatory-menstrual cycle on female athletic performance, aligning with prior investigations that have explored its effects on strength, endurance, and recovery [12,15,35]. These hormonal fluctuations necessitate training programs that account for cyclical physiological changes to optimize performance and minimize injury risk. Our recent 2025 publication [3] further advances this discourse by examining gender-specific challenges in planning strength training loads and sports preparedness for elite female athletes in team sports. Using psychophysiological and biomechanical methodologies, we identified distinct differences in balance function and psychophysiological states between male and female athletes. Notably, female athletes demonstrated superior balance function with their eyes closed, indicating potential advantages in proprioceptive control [3]. Furthermore, we confirmed that female athletes have a lower center of gravity than males, which affects biomechanical considerations, such as the optimal knee angle during barbell squats. These findings challenge conventional training guidelines, which are often derived from male-centric research, and highlight the need for female-specific recommendations [1,16,19].

Despite these contributions, our study has limitations. The sample size, although diverse, may not fully encompass all factors influencing female athletes, such as sport-specific demands, training experience, or cultural contexts, which cautions against broad generalizations. Potential biases, including accessibility constraints and variability in data collection, may have introduced inconsistencies, necessitating a cautious interpretation of results. A critical issue in elite sports is the application of male-derived training models to female athletes, particularly in speed and strength training, which can be detrimental to performance and increase injury risk [12,16]. Our analysis of expert evaluations from countries including the USA, Canada, UK, Switzerland, Norway, China, South Africa, Ukraine, and Poland revealed a global lack of attention to gender-specific training needs. This oversight underscores the importance of integrating gender differentiation into training planning to enhance effectiveness and equity.

Hormonal fluctuations significantly affect female athletic performance, necessitating advanced monitoring to tailor training protocols effectively. High-performance liquid chromatography (HPLC) offers precise steroid quantification but is impractical for routine use [42]. Mass spectrometry, while accurate, is similarly invasive and less feasible for frequent monitoring [43]. Point-of-care diagnostics, such as blood coagulation tests, highlight the potential for non-invasive alternatives [44]. Research in female sports faces unique challenges, underscoring the need for gender-specific approaches [45]. Saliva-based hormone testing offers a practical and non-invasive method for regular monitoring [46]. Graphene-based materials, including hydrogels and carbon nanomaterials, show promise for detecting steroid hormones with high sensitivity [47,48,49]. Adsorption technologies targeting 17β-estradiol further enhance monitoring capabilities [50]. Graphene’s biomedical applications, such as biosensing, enable real-time data collection [51,52]. Wearable sweat biosensors and laser-scribed graphene sensors offer innovative, non-invasive solutions for hormonal tracking [53,54]. Electrochemical carbon nanotube membranes and signal-enhanced immunoassays improve detection accuracy for steroid hormones [55,56]. Novel aptamers facilitate rapid, colorimetric detection of steroids, supporting practical applications [57]. These advancements address sex-specific performance differences, promoting tailored training and fairness in women’s sports [58].

For interim guidance, coaches can use subjective wellness questionnaires (e.g., daily RPE and mood tracking) to adjust loads: reduce volume by 15–20% during reported premenstrual fatigue. In team sports, group athletes by approximate phase clusters (e.g., 3–4 groups) for modified drills, but full individualization awaits better tools.

The core finding of this study is that none of the tested low-cost, non-invasive methods (calendar counting, basal body temperature, cervical mucus ferning, questionnaires) provide sufficiently reliable, real-time, and coach/athlete-friendly information to enable menstrual cycle-based training individualization in everyday practice—even when applied to a small, highly motivated group of elite athletes under close scientific supervision. If these methods fail even under the most favorable conditions imaginable, they cannot be expected to succeed in recreational fitness, individual sports, or less-structured team environments. Therefore, until a simple, accurate, and truly field-applicable hormonal monitoring device is developed, the wealth of scientific knowledge about cycle-related performance fluctuations will remain largely theoretical. The present results provide precise functional requirements for such a future device: it must (i) simultaneously detect at least estradiol and progesterone, (ii) deliver results within minutes, (iii) require no daily sample collection by the user, and (iv) be affordable and robust for daily training use by coaches and athletes at all levels.

The novel contribution is not the confirmation of phase effects (already known [11,59]), but the demonstration of practical implementation failures in a real-world elite setting.

## 5. Limitations

The study was conducted on a relatively small sample (*n* = 25) of elite female basketball players. This was a deliberate choice: by testing monitoring methods in the most controlled and motivated professional environment possible, we maximized the chances of successful practical implementation. The failure of these methods under such ideal conditions strongly reinforces the conclusion that the identified practical barriers are relevant to all physically active women, regardless of sport or performance level.

## 6. Conclusions

This study elucidates the critical influence of hormonal fluctuations across the menstrual cycle on the physical and technical performance of elite female basketball players, with significant performance enhancements observed during the postmenstrual and post-ovulatory phases and reductions in performance during the premenstrual phase—insights with profound implications for public health regarding promoting women’s physical and mental well-being through sustained engagement in sports. Meta-analysis demonstrates the practical application of non-invasive hormonal monitoring methods, such as urine-based devices, which showed high accuracy (95–99%), rapid results (10–20 min), and cost-effectiveness ($150–260), making them suitable for routine integration into athletic training and broader public health initiatives for female empowerment. In contrast, invasive methods such as LC-MS/MS, while highly accurate, are impractical for frequent use due to their cost, complexity, and specialized equipment requirements. The integration of psychophysiological, anthropometric, and hormonal data highlights the necessity for gender-specific training protocols that align with menstrual cycle phases to optimize performance, minimize injury risk, and address gender-based health disparities in sports participation. Current monitoring methods, such as the fern test and basal body temperature, have limitations in precision and are susceptible to external factors, underscoring the urgent need for innovative, non-invasive technologies. Advancements in biosensing technology offer promising prospects for developing sensitive, real-time monitoring solutions for hormones. These innovations could revolutionize training personalization for female athletes, address gaps in male-centric sports science, promote equity, and enhance public health outcomes by increasing female participation in physical activities and fostering inclusive wellness programs. Despite these insights, the study’s sample size and potential data-collection biases warrant cautious interpretation, and further research with larger, diverse populations is needed to validate and expand these findings. Developing accessible and reliable hormonal monitoring methods remains a critical priority to enhance performance, health, and reproductive well-being in women engaged in fitness and sports, ultimately contributing to equitable public health strategies.

Further research in off-season vs. in-season contexts is recommended.

## Figures and Tables

**Table 1:** Standard Methods for Hormone Detection.

Method	Invasive/Non-Invasive	Pros	Cons	References
Liquid chromatography-tandem mass spectrometry (LC (HPLC) with MS/MS)	Invasive (blood samples, interstitial fluid (ISF))	High accuracy (99% and more), high specificity, multi-hormone profiling	Costly, requires specialized equipment and trained personnel	[25]
Immunoassay/Chemiluminescence Immunoassay (ELISA/CLIA)	Invasive (blood samples, interstitial fluid (ISF)); Non-invasive (saliva, urine, sweat)	Simplicity, low cost, requires minimal laboratory conditions, and does not require specialized personnel skills	Lack of specificity, sensitivity, cross-reactivity with other hormones and metabolites, and matrix effects	[28]
Optical methods (fluorescence, UV, and Raman spectroscopy)	Invasive (blood samples)	Cost-effective and field-adaptable	Lack precision for quantitative analysis and are susceptible to environmental interference.	[27]
Electroanalytical (electrochemical sensors, patches, rings, etc.)	Non-invasive (saliva, urine, sweat)	High accuracy (picomolar range), portable, rapid results, continuous monitoring (via wireless data transmission)	Lower hormone concentrations compared to blood, variability due to saliva flow rates or contamination, concerns about sensor durability during intense physical activity	[27]

**Table 2:** Weekly Training and Testing Schedule.

Day	Activity	Tests Conducted
Monday	Training + Testing	Acceleration, agility, and strength
Tuesday	Training + Testing	Vertical jump, endurance, and technical precision
Wednesday	Training + Testing	Agility, power, and sprint analysis
Thursday	Training + Testing	Endurance, strength, and movement assessment
Friday	Training + Testing	Vertical jump, acceleration, and agility
Saturday	Training + Testing	Technical precision, power, endurance
Sunday	Rest	None

**Table 3:** Meta-Analysis of Hormone Detection Methods.

Method	Trademark	Sampling	Accuracy	Fastness	Price (USD)	Simultaneous Estrogen + Progesterone
ELISA/CLIA	Cerascreen	Saliva	87.50%	7 days	100	±
LC-MS/MS	Walk-In Lab	Saliva	>99%	7–21 days	300	+
ELISA/LC-MS	LifeLabs	Blood	85%	7–10 days	80	+
ELISA	Mira	Urine	99%	20 min	260	−
ELISA	Inito	Urine	99%	10 min	150	−
ELISA/CLIA	DRG International	Saliva/blood	85–90%	2–4 h	150–200	+
ELISA/CLIA	IBL International	Saliva/blood	90–95%	2–4 h	200–250	+
ELISA	Abcam	Saliva/blood	90–95%	2–4 h	200–300	+
ELISA	Salimetrics	Saliva	92–96%	2–4 h	180–250	−

Note: + means yes: The method/product fully supports simultaneous detection of both hormones (e.g., in one sample, one test strip, or one panel/kit). − (minus sign) means no: The method/product does not measure both hormones together; it may measure one (often estrogen/LH for ovulation prediction) but not progesterone, or requires separate tests. ± means partial or variable: The method/product can measure both in some configurations, kits, or optional add-ons, but not always standardly or simultaneously in the base/single test (e.g., estrogen is core, progesterone is available separately or in certain bundles).

## Data Availability

The data presented in this study are available on request from the corresponding author due to (the data are not publicly available due to privacy or ethical restrictions).

## Abbreviations

ANOVA	Analysis of Variance
BBT	Basal Body Temperature
CLIA	Chemiluminescence Immunoassay
E2	17β-Estradiol
E3G	Estrone-3-Glucuronide
ELISA	Enzyme-Linked Immunosorbent Assay
FSH	Follicle-Stimulating Hormone
HPLC	High-Performance Liquid Chromatography
IA	Immunoassay
IoT	Internet of Things
ISF	Interstitial Fluid
LC-MS/MS	Liquid Chromatography-Tandem Mass Spectrometry
LH	Luteinizing Hormone
LOD	Limit of Detection
MC	Menstrual Cycle
P4	Progesterone
PdG	Pregnanediol Glucuronide
PCOS	Polycystic Ovary Syndrome
TMB	3,3′,5,5′-Tetramethylbenzidine
UV	Ultraviolet
